# Development of real world learning opportunities in community exercise prescription for healthcare professional programmes - ‘Physio Hub’

**DOI:** 10.1186/s12909-021-02503-3

**Published:** 2021-01-26

**Authors:** Caitriona Cunningham, Catherine Blake, Grainne O Donoghue, Ciaran Purcell, Ulrik Mc Carthy Persson, Karen Cradock, Sinead Mc Mahon

**Affiliations:** School of Public Health, Physiotherapy and Sports Science, University College Dublin, Belfield, Dublin 4, Ireland

**Keywords:** Clinical practice education, Curriculum development, Exercise prescription, Exercise is medicine, Health promotion, Healthcare professional education, Physiotherapy, Service learning

## Abstract

**Background:**

Given the challenge of chronic lifestyle diseases, the shift in healthcare focus to primary care and recognised importance of a preventive approach to health, including exercise prescription, the embedding of related learning in healthcare professional programmes is critical.

**Methods:**

In response to these contemporary demands, a complex curriculum development project was undertaken at University College Dublin, employing a four dimensional curriculum framework for the development of health professional curricula, that focused on (1) future orientation of healthcare practices (the why?), (2) defining capabilities of graduates (the what?), (3) teaching, learning and assessment (the how?) and (4) organisation/institution delivery (the where)? The process was informed by latest exercise, health promotion, educational and health policy literature, alongside engagement with multiple internal university and external community stakeholders.

**Results:**

Having sufficient clinical education opportunity for translating exercise theory into practice was identified as a key need (the Why?). Development of strategies for health promotion and design and delivery of evidence based exercise programmes with inter-professional and inter-sectoral network building were some of the graduate capabilities identified as being critically important. (the what?) The resultant UCD Physio Hub model of clinical education combines ‘on campus’ and ‘community outreach’ activity to facilitate inter-sectoral ‘real world’ experiential student learning in health promotion and exercise prescription for both healthy and clinical populations. Underpinned by social constructivist educational theory, students are encouraged to be creative and to collaborate in responding to identified health needs of specific community groups by designing and delivering community services. (the how?) In developing new student learning opportunities to enhance curriculum, a supportive organisational culture and context was critical with UCD having excellent exercise infrastructure and the Physio Hub project aligning with a community engagement ethos articulated in the university’s strategy. (the where?)

**Conclusion:**

This paper provides an overview of Physio Hub, its services, educational practices and translational research ethos, all of which are combined to deliver a rich exercise and health promotion learning experience. Although developed for physiotherapy in this instance, the curriculum process and resultant education model could be applied across medical and other health professional programmes and to facilitate interdisciplinary learning.

**Supplementary Information:**

The online version contains supplementary material available at 10.1186/s12909-021-02503-3.

## Background

Any effort to change a healthcare system requires focus on the education of future clinicians who will practice new approaches in new contexts [1] with reconsideration of traditional health professional **clinical education** models required [2]. Fostering learning in primary and community health promotion and engagement [3, 4] with greater emphasis on positive health behaviours including exercise and physical activity (PA), as highlighted by the Exercise is Medicine initiative, is critical to address the current lifestyle and chronic disease crisis [5]. Systematic embedding of physical activity and exercise theory and practice in entry level healthcare professional education programmes is necessary [3, 6–10] and key learning domains have been identified [11]. Given the broad recognition of the social determinants of health and health behaviours [12, 13], diversification of clinical education to include less traditional community settings, schools, sports clubs, health promotion units and tertiary centres, allowing critical inter-sectoral, prevention and management of lifestyle related disease experience across diverse populations is warranted [14]. This will allow educators to purposefully engage with learners in direct experience and focused reflection, developing student capacity to contribute to their communities with a view to shaping future professional practice [15, 16].

In order to reflect such contemporary societal health needs, ongoing curriculum review and enhancement projects are required, as happened within the Physiotherapy discipline at University College Dublin. Accompanied by a curriculum enhancement research programme, this resulted in a number of significant curriculum innovations at UCD, including the establishment of ‘Physio Hub’, a novel clinical education model. This new model facilitates inter-sectoral ‘real world’ student learning in community health, physical activity and exercise prescription for both healthy and diseased populations.

Establishing Physio Hub was a complex project, involving multiple stakeholders and given the numerous and often competing demands on educators and health systems a rigorous and comprehensive conceptual framework was adopted [17]. This should facilitate replication of the Physio Hub curriculum initiative,wholly or in part, at other higher education institutions (HEIs) and across disciplines.

Th aim of this paper is therefore to describe the curriculum development process that led to UCD ‘Physio Hub’, using a curriculum development framework, providing institutional context, underlying pedagogy, an overview of content and delivery mechanisms and preliminary data relating to its evaluation.

## Methods

### Curriculum development- project setting

University College Dublin (UCD) is one of Europe’s leading research-intensive universities and Ireland’s largest University with a longstanding reputation in providing healthcare professional (HCP) education, including Medicine, Physiotherapy, Nursing, Midwifery, Diagnostic Imaging, Clinical Psychology, Social Work, and Clinical Nutrition. In addition, UCD is nationally renowned for its sports and exercise facilities, attracting the country’s leading athletes through its sport scholarship and elite athlete training academy. The UCD School of Physiotherapy (established 1955) offers both BSc (4 year) and MSc (2 years for graduates) entry level Physiotherapy programmes (280 fulltime students), alongside post graduate taught specialist and research programmes.

### Curriculum development framework approach

**Fig. 1:**
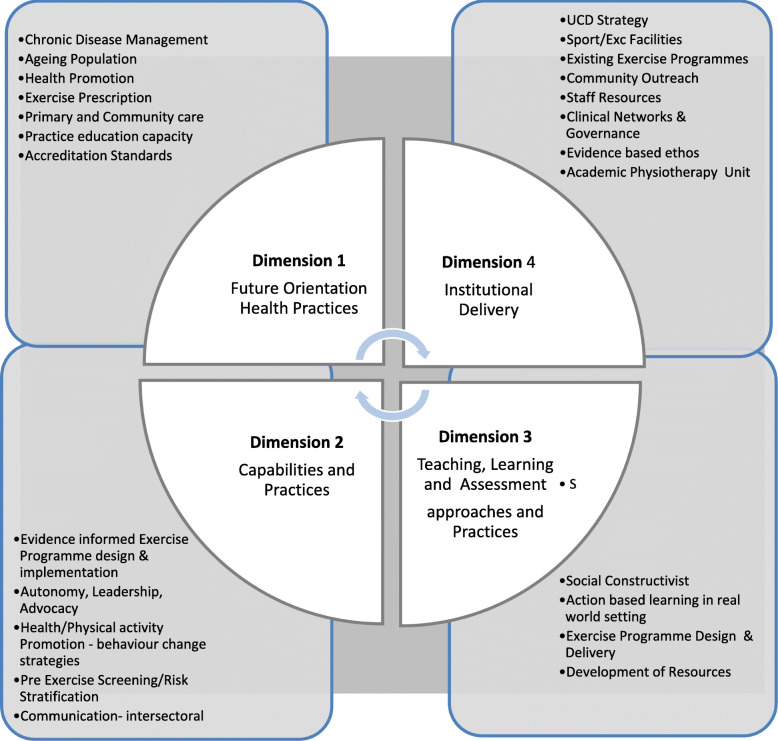
UCD Physio Hub Clinical Education Development Process - 4 D Framework [17]

Steketee et al’s [17] ‘four dimensional framework for the development of health professional curricula’ was adopted as the scaffold for this complex curriculum project, the focus of which is to provide experiential learning in community health, physical activity and exercise prescription for healthcare professional students. This framework was selected as it recognises the dynamic, multi-dimensional and integrated nature of curriculum and the need to connect health directly to the larger political, social and economic issues [17]. It is comprised of four key dimensions to frame curriculum development projects: (1) future orientation of healthcare practices; the why? (2) defining capabilities of graduates; the what? (3) teaching, learning and assessment; the how? and (4) institutional delivery; the where? Figure 1 provides an illustration with framework dimension detail relevant to the current project.

### Physio hub Curriculum development project team

A ‘Physio Hub' curriculum development project team’ was established, comprised of university academic and clinical education staff with education, clinical and research expertise in the following areas: physical activity and exercise prescription, public health, health promotion, healthcare professional clinical education and curriculum design and development. All were registered healthcare professionals.

### Stakeholder involvement

University stakeholders can be classified broadly into two distinct groups: curriculum stakeholders and professional stakeholders [18] with students and health service users recognised as important curriculum stakeholders [19] and many educators often having dual roles on healthcare professional programmes with parallel or prior clinical practitioner roles. In the case of this project, education and exercise researchers (CC, GOD, SMcM) all co- authors on this paper engaged in a series of formal studies which underpin this project. (Table 1).

**Table 1:** Physiotherapy Curriculum Research (UCD) informing Development of Physio Hub

Author & Year	Title	Data Sources	Key Findings -informing development of Physio HUB
**O’Donoghue,** G., Doody, C., & Cusack, T. (2011^a^) [20].	*Physical activity and exercise promotion and prescription in undergraduate physiotherapy education: content analysis of Irish curricula*	Content Analysis All physiotherapy curricula in Ireland	There is a need for **re-evaluation and enhancement** of **Physiotherapy curricula** in Ireland to align to public health & lifestyle related disease in relation to physical activity and exercise
**O’Donoghue** G, Doody, C., Cusack T (2011^b^) [21].	*Using student-centred evaluation for curriculum enhancement: An examination of undergraduate physiotherapy education in relation to physical activity and exercise prescription*	Structured Group Feedback Sessions All Physiotherapy students in Ireland (*n* = 135)	Course content, **Practice Placement**s and Teaching and Learning methods were the identified theme (required enhancement areas)
**O’Donoghue** G,Cusack T., Doody C (2012) [22].	*Contemporary Undergraduate Physiotherapy Education in terms of Physical Activity and Exercise Prescription: Clinical Tutors’ Knowledge, Attitudes and Beliefs*	Questionnaire & Focus Groups All physiotherapy practice tutors In Ireland (*n* = 38)	Practice Tutors identified a need for **further education** and training to improve their knowledge base in relation to exercise prescription
**O’Donoghue** G, Aagard-Hansen J, Murphy F, Woods C, **Cunningham C** [10].	*Assessment and Management of Lifestyle Risk Factors for the Prevention of Type 2 Diabetes and Cardiovascular Disease: A SNAP -shot of Physiotherapy Primary Health Care Capacity in the Republic of Ireland.*	Survey Primary care Physiotherapists (*n* = 220)	**Primary care physiotherapists are a key resource in terms of assessment of lifestyle risk management**, one that is under used in current health care systems.
**McMahon, S.**, Cusack, T., & **O’Donoghue, G**. (2014^a^) [23].	*Barriers and facilitators to providing undergraduate physiotherapy clinical education in the primary care setting: a three-round Delphi study.*	3 round Delphi Survey Practising physiotherapists & managers in primary care in Ireland (*n* = 198)	The **need for primary care placements** is acknowledged. Clear planning and **collaboration with all stakeholders** the main barriers could be addressed
**McMahon, S.,** Cusack, T., Waters, N., & **ODonoghue, G.** (2014^b^) [24].	*A Profile of Physiotherapy Practice Education settings 2009–2012. Physiotherapy Practice and Research, 35, 95–100*	Survey of all physiotherapy placement co-ordinators in Ireland (*n* = 4 at 4 HEIs)	**Only 5% of all physiotherapy placements** were in primary care over a three year period.
**McMahon, S., O’Donoghue, G.,** Doody, C., O’Neill, G., & Cusack, T. (2016^a^) [25].	*Expert opinion regarding the preparation of entry-level physiotherapists for primary healthcare practice, examined using Biggs 3P’s model of teaching learning*	Semi-structured Interviews – experts in primary care/ education (*n* = 12)	Understanding the philosophy of primary healthcare and **the role of health promotion as a key to primary care practice** was among the themes identified
**McMahon S, O Donoghue,G**., Doody C, O’Neill G, Cusack T (2016^b^) [26].	*Standing on the precipice – Evaluating final year physiotherapy students’ perspectives of their curricula as preparation for primary healthcare practice*	Structured Group Feedback Sessions Final year Physiotherapy students (*n* = 68)	**Lack of primary healthcare placements** was one of the main issues identified by students

To ensure broad representation, in terms of curriculum mapping to contemporary healthcare needs and defining graduate attributes for diversification of employment opportunity, a multi-stakeholder consultation process was instigated. This included drawing on the curriculum team’s existing collaborations and co-opting new stakeholders to include both internal (academic, clinical, clinical education staff and students) and external stakeholders (clinicians, community organisations). Other key stakeholders included University management, legal, health and safety, quality and data protection representatives to ensure appropriate governance arrangements. The consultation process involved a series of dedicated group and one-to-one meetings, e-mail and phone communications with the wider stakeholder group over a 6 month period. Table 2 provides an overview of stakeholder key meetings and workshops with a timeline and deliverables.

**Table 2:**
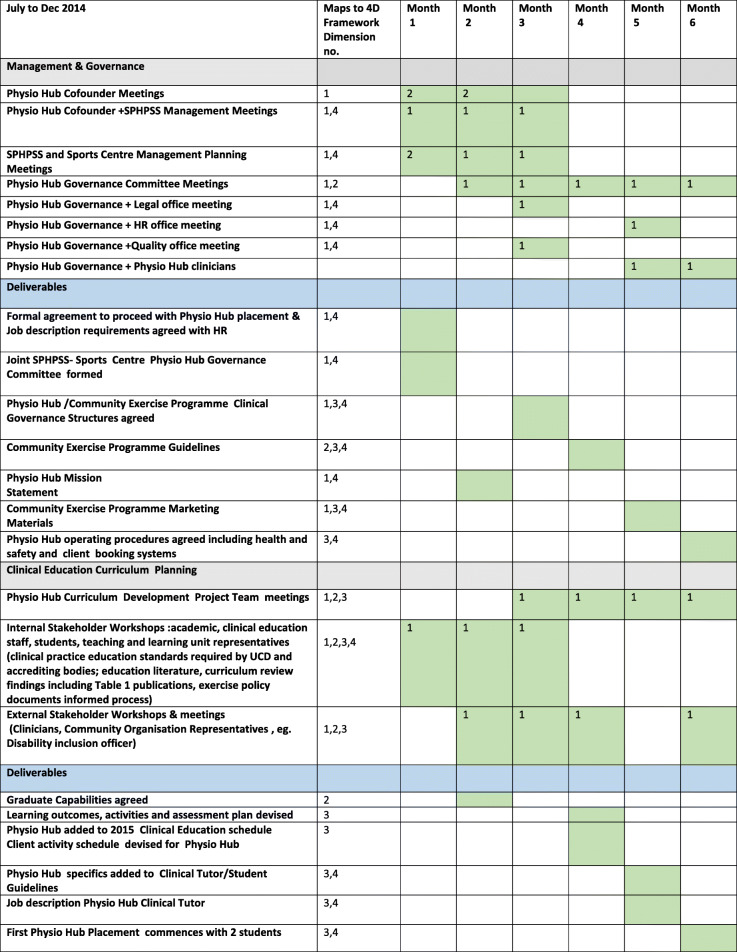
Physio Hub Project: Meeting Schedule and Deliverable Timeline Overview

Physio Hub Governance Committee: Physio Hub Cofounders (CC, SMcM, CB) + Sports Centre Management

Physio Hub Curriculum Development Project Team: Physio Hub Cofounders (CC, SMcM, CB) + GOD, UMcC + Clinical Education Team

Physiotherapy Academic Staff (CC, CB, UMcC, GOD) + students (*n* = 5) + Clients (*n* = 3)

Clinical Education Team (SMcM, CP,KC);Sports Management Team: Sports Centre Manager, Administrator, Sports Service Development Officer

Clinicians (Physiotherapists *n* = 5,Medical *n* = 3, Nursing *n* = 3, Dietetics *n* = 2,Pharmacist *n* = 1);Community Organisations Representatives (*n* = 3)

## Results

The process of curriculum development, resultant Physio Hub and preliminary evaluation represent the results of this paper.

### Dimension 1. Future orientation of healthcare practices: the why?

In the first dimension, the focus was on ‘the why’? Why is this novel model of clinical education needed? Whilst healthcare professional curricula fittingly respond to the requirements of registration and accreditation bodies [27, 28] it is imperative that they be responsive to service demands and shaped through work-based, inter-professional, inter-sectoral and public health foci [17]. Ireland as elsewhere is facing unprecedented health system challenges with an ageing population and a high prevalence of chronic, lifestyle diseases, demanding a shift in the focus of health care from an acute hospital service model, which treats disease, to a service which focuses on prevention and health promotion in community care settings [23, 25, 26, 29–33]. Acknowledging that University and health system based learning opportunities may differ between Higher education institutions in Ireland, in the 3 year period of 2009 to 2012, only 5 % (*n* = 171/3142) of all physiotherapy placements in Ireland were in primary healthcare settings [24] with a lack of student community health promotion and exercise education opportunities in the primary healthcare setting [20] (Table 1). Despite progress in appointing more Primary care teams in Ireland, significant shortfalls persist in terms of students gaining the relevant clinical experience and acute hospital approaches continue to dominate within health systems [22, 25, 34, 35]. Recent national chronic disease curriculum project work [36, 37] revealed a lack of experiential learning opportunities, for the recommended physical activity promotion interventions, within healthcare professional curricula.

Whilst exercise related learning occurs across a continuum of theory (eg. Exercise physiology), clinical skills and practice education modules, our curriculum review identified that it was in the domains which focus more on translating exercise prescription into practice (clinical practice education) that gaps existed [20, 21] (Table 1). This evidence base, in combination with a shortage of practice education opportunities in community settings, the changing employment landscape for many HCPs and ongoing stakeholder feedback, were the key drivers for the ‘Physio Hub’ initiative. A mission statement was agreed that encapsulates why the ‘Physio Hub’ was established, what it stands for, what it considers to be its fundamental purpose and its overall ethos;

“*To optimise the health of our students, staff and the wider community through the provision of evidence informed community health promotion and exercise services, led by health professionals and supported by health professional students. Underpinning this is a philosophy of integrating service provision with student education and research, consistent with best international practice”*

### Dimension 2. Defining graduate capabilities: the what?

Dimension 2 was concerned with identifying learning outcomes, expressed in relation to standards and sets of attributes, knowledge, skills and capabilities as well as dispositions. Health professional practice is multidimensional, contextually specific and relationally complex, and this must be reflected in the capabilities of graduates [17]. Although UCD’s HCP programmes have clearly articulated graduate capabilities, mapping to international professional standards, the need to reflect the changing healthcare landscape demanded that the curriculum review team identify additional capabilities specific to community healthcare and exercise prescription. More generic graduate attributes of autonomy, leadership and advocacy coupled with those more specific to community health promotion and exercise prescription were identified as important by way of the stakeholder consultation process, literature review, professional and accrediting body position statements and competency documentation [6, 11, 20, 37–40]. Clinical exercise physiology, cellular and systemic implications of exercise, health behaviour change, physical activity and public health and integrating PA and exercise into health systems were identified as key exercise learning domains for healthcare professionals. Stakeholders identified that other capabilities, including development of strategies for exercise promotion and building inter-professional and inter-sectoral networks among exercise and healthcare professionals are often more challenging to address at traditional clinical education sites.

Key learning outcomes articulated for students include achievement of competence in: assessment of physical activity levels, pre exercise screening and risk stratification, exercise programme design, delivery of safe and evidence informed exercise programmes, creation of exercise and health promotion resources for clients, interaction with clients (individual and group) which reflects behaviour change theory, measurement of outcome, client advocacy, leadership, interdisciplinary and inter-sectoral communication skills and administrative aspects of integrating physical activity in health systems.

### Dimension 3. Teaching learning and assessment: the how?

**Fig. 2:**
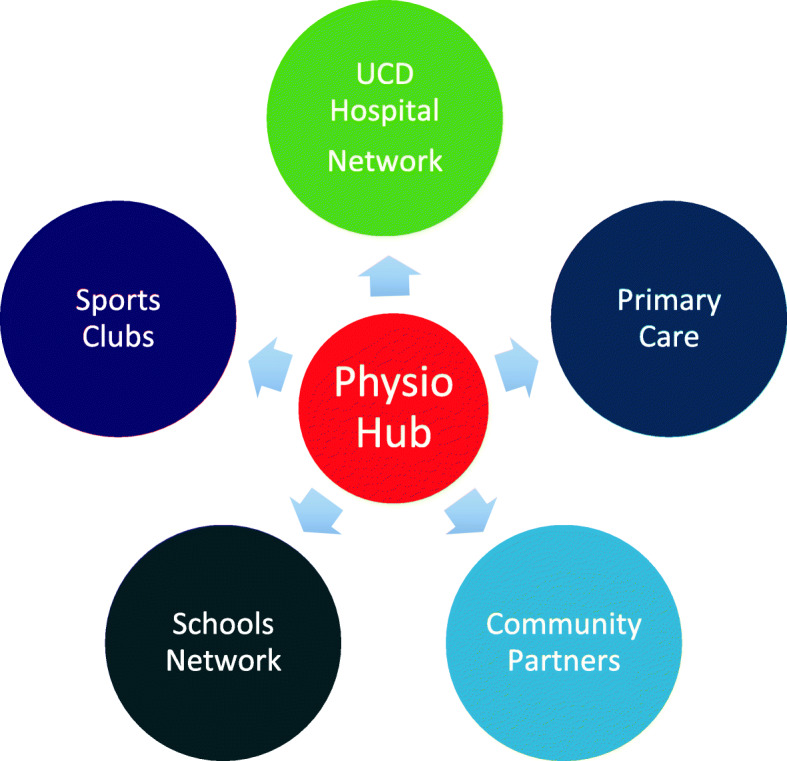
UCD Physio Hub – On Campus and Outreach Services

Teaching, learning and assessment, core to higher education, make up the ‘the how’? and are the focal point of dimension 3. Research conducted by the current authors (Table 1) and the stakeholder consultation process revealed the limitations of learning about exercise in the classroom context, the value of contextual learning [41] and the need to provide a more focused opportunity to facilitate theory-practice transfer [42]. The social determinants of health and health behaviours [12, 13] are well recognised, including the interpersonal, social and environmental elements required to facilitate client PA and exercise [43]. Thus, creating a clinical education environment with a socially mediated context such as a community gym to facilitate exercise learning was agreed on as an appropriate and realisable solution for the curriculum project team. The ‘Physio Hub’ is therefore located within UCD Sport (university sports complex) where it acts as a community health hub with a focus on exercise prescription for healthy sedentary and clinical populations and sports injury prevention both on campus and via outreach activity with community and health service partners (Fig. 2). Initially it built upon a small number of existing physiotherapy led community exercise programmes based at UCD Sport but a further client base has been achieved by embarking on projects to increase inter-professional and inter-sectoral working, that serve both the university and wider community. Physio Hub services see health promotion and exercise prescription being realised for healthy and clinical populations across the lifespan, making student experiential learning in these areas a reality for our students (listed and described in Table 3). A university appointed physiotherapy clinical educator supervises students with the emphasis on facilitating self -directed learning (average of 1:5 as tutor: student ratio), using a a mix of direct, indirect and remote supervision, encouraging students to become confident in their practice, while assured that advice and mentorship is always available.

**Table 3:** Physio Hub Exercise and Health Promotion Service Provision Summary

Services/ Programmes	Target Groups	Overview
Get in Gear/Active Campus Europe^a^	Sedentary University Students	Health screening and exercise programme × 12 weeks aligned with academic terms
UCD Better Bones^a^	Age 55+ with bone health issues/osteoporosis	Group Exercise and interdisciplinary education programme × 12 weeks on campus
UCD Better Hearts^a^	Clients with cardiac health issues	Group Exercise and education programme × 12 weeks on campus
Injury Prevention Sports Teams^a^	UCD/local club Gaelic Athletic Association football/hurling, UCD Soccer, Rugby	Injury Prevention Programme over season at UCD/other local clubs
Crumlin Olympic Challenge	Children with intellectual Disability, their teachers & parents	6 weeks- health promotion talks & exercise classes both in community and at UCD sport
UCD-Simmons College Boston, USA Traveller Healthy Living Project	Traveller Community Pavee Point Traveller Representative Organisation	International Service Learning Health Promotion project
Enable Ireland-UCD Kids’ Camp	Children with Physical Disability	Physiotherapy supported kids’ multisport camps on campus
Parkinson’s ‘On the Move’	Individuals with Parkinsons’ Disease	12 week exercise programme (Spin/Circuit/Tai Chi) with related research on campus
UCD-DLR Rathdown Leisure services ‘Move don’t Sit’ (MedaliST)	Older adults (> 65) with multiple morbidities in area of social disadvantage	12 week exercise & public lecture series at community gym
Healthy Eating Active Living Community Health Project	Primary School Children	Fitness testing, PA programme & health promotion talks at primary school (6 weeks)
UCD Festivals	UCD and Local Community	Fitness testing & PA promotion at hub health & wellbeing festival zone
WCPT Active Ageing	Older Population	Exercise Class & Public Lecture on campus
Darndale Active Girls Project	Teenage girls in area of social disadvantage	Social entrepreneurship PA promotion project
UCD Workplace Health	Office workers on Campus	Development of Guidelines for Workers

Note: List not exhaustive; each practice education block has an embedded active learning project and some initiatives are threaded through all practice education blocks, with modifications as required

^a^Longstanding programmes running several years providing consistent client base

The underlying pedagogy is one of social constructivism [44] with a focus on advancing psychomotor, clinical reasoning and metacognitive skills and recognition of the social and environmental facilitators of exercise behaviours. Consistent with this pedagogical approach, students at the ‘Physio Hub’ are encouraged to lead, collaborate and negotiate with one another, their clinical educator, service-users and community partners, to be creative and to co-construct knowledge and resources in order to develop, market, deliver and formally evaluate research- informed exercise and community health initiatives in a socially mediated context. This learning is facilitated by embedding an action based learning project within their educational experience to address real world health challenges [45]. Projects have included delivery of health promotion and exercise initiatives on campus that target the general population and community outreach activities that assist students’ inter-sectoral learning in community organisations including disability organisations, schools, sports cubs and local authority fitness facilities. Table 3 provides more detail. Students are encouraged to leverage technology in preparation for the rapidly changing and networked world of work (e.g. fitness apps, wearable sensor technology, social media) [46]. Consistent with its translational research ethos, the ‘Physio Hub’ acts as a ‘living laboratory‘to support exercise related research, facilitating BSc, MSc and PhD level research projects with students assisting in data collection, data entry and analysis. Standard assessment of student performance is conducted using the common assessment form (CAF) for Physiotherapy practice education with multiple learning outcomes grouped into five domains (Assessment, Treatment, Professionalism, Documentation and Communication) [47] and with additional assessment criteria utilised to reflect identified capabilities (Dimension 2) and related learning outcomes. Novel assessment strategies are embedded over the 6 week Physio Hub experience to drive learning that achieves the more context- specific learning outcomes. These include design and delivery of; exercise programmes, online resources, brief interventions, public health talks and presentations to multi-sectoral audiences regarding proposed new exercise services and their evaluation. Impact on student learning is captured by way of assessment rubrics which incorporate both generic and more specific exercise and health promotion learning outcomes in combination with student reflective learning portfolios and formal placement feedback surveys.

### Dimension 4. Supporting institutional delivery - the where?

In developing new student learning opportunities to enhance curriculum, the organisational culture and context is crucial to the process. The Physio Hub project aligned well with the university strategy (Additional file 1), providing an experiential learning environment to strengthen scholarship in disciplines, facilitate translation of theory to practice, build engagement locally and nationally, service student health well -being and sport and with demonstrated ability to attract international students via unique service learning opportunities [45]. UCD has a programmatic approach to curriculum design and enhancement with emphasis on learning outcomes and graduate attributes and with health professional programme curricula needing to map to professional accrediting and state registration body requirements [27, 28]. The top class exercise facilities at UCD, coupled with a history of positive working relationships and resource sharing between the physiotherapy academic, sport and leisure service units and clinical networks, was a key enabler with this ecosystem acting as a springboard for the new initiative. This project copper fastened shared goals and built the case for the redeployment of space to the Hub, leveraging of the existing client booking system, reception services and also linking into additional channels for promotion and ‘branding’ of the Hub activities*.* **Governance, management and budgetary structures to enable the team’s vision were realised via consultation with representatives from** UCD quality, legal, data protection, health and safety and existing clinical partners (Table 2). This resulted in the formation of a Physio Hub governance committee comprised of representatives from the UCD academic physiotherapy unit (CC, SMcM, CB) and UCD Sports Centre with a joint clinical governance arrangement between UCD and its clinical partners.

### Preliminary evaluation

Although the focus of this paper is on the process of curriculum development for UCD Physio Hub, some preliminary outcome evaluation has been conducted from both the student and institution perspectives.

### Student performance and feedback

Levels of student engagement are excellent as evidenced by students rising to the challenge of addressing real world issues and excelling in delivering projects with tangible outputs (Table 3). Student grades achieved for the Physio Hub practice education module indicate a very strong student performance and verify the achievement of the National Common Assessment Form [47] learning outcomes which underpin the University’s practice education modules and are required by accrediting bodies. Student feedback on all clinical practice education and other academic modules at UCD is standard and student feedback from the Physio Hub has been utilised on an ongoing basis to inform enhancement of the learning experience. Overall feedback on the unique gym- based learning experience is very positive, although adapting to the less traditional and more self directing nature of this unique placement requires a shift in student thinking in the early stages of placement. UCD Physiotherapy students rank Physio Hub in their top five practice placement choices of 60 potential options.

In addition, online student surveys were conducted with all students, who completed a 6 week placement at Physio Hub from May 2019 to October 2020 (*n* = 28) and consented to data reporting. Students were asked to rate their level of agreement (1 to 5) with a series of statements relating to achievement of learning outcomes informed by graduate capabilities (Dimension 2). Outcomes where > 80% of students reported achieving the learning outcome included: appraisal of exercise evidence, health promotion skills, exercise programme design and delivery, behaviour change communication skills, team work, collaboration, inter sectoral communication, producing written reports, chairing meetings and time management skills. In addition, formal assessment of the embedded ‘real world’ action learning projects over multiple placements demonstrates students’ achievement of both planned and unplanned learning outcomes including; enhanced skills in community exercise prescription, inter-sectoral communication, project management, time management, advocacy, team work, exercise programme administration, marketing and design of health promotion resources.

### Institutional benefits

From an institutional standpoint, the Physio Hub has had many benefits supporting UCD’s stated goal of maximising relevance and impact on society, while at the same time augmenting the student learning experience and researcher engagement (Additional file 1).

Physio Hub has added significantly to practice education capacity, a major challenge for higher education institutions. To date, 140 BSc and MSc (graduate entry) physiotherapy students have completed blocks (4 to 6 weeks each) of professionally accredited practice education at the Physio Hub. The Hub placement started with only two students, whereas currently twenty six students are given the opportunity to complete a placement at the Hub each year. This represents 7% of the overall placement requirement for the physiotherapy programmes at UCD and with potential for expansion with further inter sectoral, community partnerships.

Physio Hub has enabled UCD Physiotherapy students to contribute both to the University community and broader society aligning with service learning models [48]. The exercise and health promotion programmes delivered all represent examples of community engagement that would not otherwise occur (Table 3) with client contacts now in excess of 2200 per annum. Strategic partnerships with public and non-governmental agencies, education, community and professional organisations have been established, consistent with both a public engagement and community outreach ethos. Physio Hub initiatives map well to the ‘Healthy UCD’ staff and student well-being initiative and were integral to UCD achieving ‘Exercise is Medicine’ campus accreditation in 2020.

### Formal recognition of the Physio Hub as an education initiative has been achieved as follows

- International ‘Exercise is Medicine’ Campus Accreditation Silver Award (Exercise is Medicine, 2020)
- European Network of Academic Sports Services Competitive Award (2018) (Students helping Students category) for Active Campus Europe Project
- UCD Teaching and Learning Competitive Award -‘Outstanding Contribution to Student Learning’ (2018)
- UCD Sustaining Partnerships Realising Change Award (2017) - Crumlin Olympic Challenge Project
- Dun Laoghaire Rathdown County Council MEDALIST programme funding to support a partnership (UCD staff and students & DLR) approach to exercise programme development and delivery for older adults in an area of social disadvantage

## Discussion

This paper describes the concept and development process for UCD Physio Hub, a unique clinical education model which provides focused real world learning in community health promotion and exercise prescription, mapping to society’s broader health priorities of chronic disease prevention and management. Emphasis on supporting and empowering individuals to live well in their own communities through encouraging individuals to engage in preventive health behaviours, including exercise, is consistent with the shift in healthcare focus to primary care. This new translational research and learning environment creates rich student learning opportunities and acts as research infrastructure to support a parallel clinical exercise research programme at the university. Additionally, the Physio Hub represents a solution for higher education institutions, who are often challenged by the finite practice education capacity of health systems, over which they have limited control and where providing the ideal range of clinical experiential learning opportunities is difficult.

The need to report the curriculum development process [49] has been addressed, using Steketees’ four dimensional curriculum framework to provide the institutional context, underlying pedagogy and an overview of content and delivery mechanisms. This enabled a shared vision of what it is that the Physio Hub set out to achieve and demanded consideration of bigger picture issues, overcoming a tendency to regard curriculum review in terms of content and delivery methods only [49]. The feasibility of the sports facility-based Physio Hub as a model of community health promotion has been demonstrated with the university thus having acted as a think-tank developing and testing a new model which may prove worthy of adoption in healthcare systems. Findings from the Physio Hub project reported here should inform the efforts of others who wish to develop equivalent initiatives at their HEI, with the evaluative act about generating reliable, valid and useful information for curriculum developers seeking to adapt programmes in the light of evolving contexts [50]. Encouraging students to gain relevant inter sectoral experience was integral to the Physio Hub project, given that most of the social determinants of health lie outside the sphere of the health sector and collaboration with governmental and non-governmental sectors is important to improve health equity [14]. Positive relationship building both internally and with external community partners was imperative in the establishment of new initiatives and our experience supports the recommendation of establishing fewer but more significant and sustainable inter sectoral partnerships which balance student and service user needs.

[35]. The service learning practices at Physio Hub map well to the well recognised PARE (Preparation, Action, Reflection, Evaluation) model of service learning [51] which represents a useful scaffold for development of further community exercise and health promotion opportunities. Learnings from the curriculum development process include the importance of having high level institutional support and leveraging the broader, education, research and governance expertise in addition to the more obvious health discipline input (Table 2).

Although student-led community-based placements appear to impact positively on the well-being of community service users and feedback from clients has been positive, more formal inclusion of patient representative groups in the curriculum development process could have taken place, consistent with patient as partner engagement approaches [19, 52]. Challenges encountered in this project related to ensuring robust clinical governance structures in a non- healthcare setting, acquiring adequate resourcing of clinical educator staff from the HEI budget, managing student expectations regarding a non traditional clinical education model and resourcing the significant administrative workload associated with developing and delivery of health services. Although this paper’s purpose was not to evaluate the student experience, this is obviously a key outcome of any curriculum development process and preliminary insights, based on initial data analysis, have revealed a positive impact on student learning. From an educator perspective, a greater level of autonomous practice has been observed with students operating as ‘portfolio’ workers, gaining valuable experience to enhance their entrepreneurial skills and career options for the current employment landscape characterised by its flexibility in working hours, freelancing, freedom in the choice of work and independent contracts [53]. Such capabilities are required of future healthcare professionals to pre-empt and be responsive to society’s healthcare needs and for enhancing career opportunities as independent contractors and advanced practitioners [54]. Further systematic evaluation of the data relating to student experience and impact on learning is already underway to ensure these critical perspectives are robustly evaluated without bias.

Models based on the Physio Hub could be implemented across a range of healthcare professional programmes either wholly or in part, dependent on the articulated discipline- specific or interdisciplinary graduate capabilities and cognisant of competing demands in more crowded curricula. For instance, medical programmes may wish to place more focus on pre exercise medical screening, risk stratification and practising delivery of brief PA interventions [37]. This may better reflect the likely medical role where onward referral to physiotherapists and other exercise professionals for development, actual delivery and monitoring of exercise programmes would occur. A shorter clinical affiliation using the Physio Hub model to achieve specifically defined learning outcomes may therefore be sufficient. The main 4D framework [17], other exercise and PA focused frameworks (see Dimension1) and guideline documents identified in this paper [6, 20, 36, 40] support transferability to other programmes and HEIs, with further recent publications supporting the drive to embed PA and exercise content in medical curricula [55–57].

## Conclusion

This paper describes a successful theoretical framework- based curriculum development process for embedding real world exercise and health promotion experiential learning opportunities in healthcare professional programmes with tangible benefits at both student and higher education institutional levels. Findings from this process have important implications for healthcare professional programme providers given the current emphasis on the need for preventive health approaches, including exercise, to be systematically incorporated in healthcare professional education programmes with opportunity for students to translate theory into practice.

## Supplementary Information

**Additional file 1.** Academic, Research and Community Goals of Physio Hub@UCD Sport: Mapping to University Strategic Objectives

## Data Availability

We the authors agree to comply with journal’s copyright and data availability guidance and are open to data requests.

## Abbreviations

4D: Four dimensional
CAF: Common Assessment Form
HCP: Healthcare Professional
HEI: Higher Education Institution
PA: Physical Activity
UCD: University College Dublin
