# Supplementary material for: Development of real world learning opportunities in community exercise prescription for healthcare professional programmes - ‘Physio Hub’
Source: BMC Med Educ. 2021 Jan 26;21:76. doi: 10.1186/s12909-021-02503-3 (PMC7836499; doi:10.1186/s12909-021-02503-3)
Supplement: Supplementary file 1 — Additional file 1. Academic, Research and Community Goals of Physio Hub@UCD Sport: Mapping to University Strategic Objectives [file 12909_2021_2503_MOESM1_ESM.docx]

Appendix 1

**Academic, Research and Community Goals of Physio Hub@UCD Sport: Mapping to University Strategic Objectives**

|  | **UCD Objectives** | Physio Hub |
| --- | --- | --- |
| 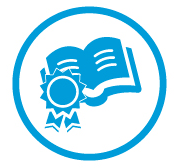 | **Increase quality, quantity and impact of our research, scholarship and innovation** | Captialise on opportunity for a living teaching and research laboratory at UCD.  Build research capacity and outputs |
| 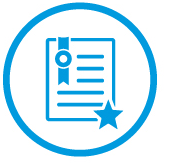 | **Provide an educational experience that defines international best practice** | Unique offering in Physiotherapy education in Ireland, incorporating translation of evidence in practice and service learning opportunities in line with international best practice, Excellence in student experience.  Future benefits for other disciplines. |
| 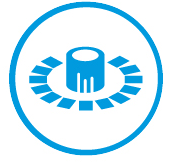 | **Consolidate and strengthen our disciplines** | Strengthens translation of theory to practice and disciplinary research impact . Provides experiential learning environment which strengthens scholarship in education and health disciplines |
| 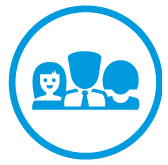 | **Attract and retain an excellent and diverse cohort of students, faculty and staff** | Unique marketing opportunity to attract overseas graduate and study abroad students : service learning critical for overseas students. |
| 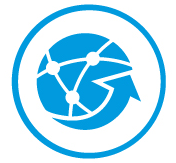 | **Build our engagement locally, nationally and internationally** | Service UCD student and staff health and well being needs with outreach to the wider community  Community partnership approach to projects increases engagement |
| 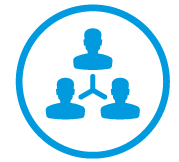 | **Develop and strengthen our University community** | Support our student and staff community. Promotion of UCD Healthy Campus  Support UCD sports teams  Active Campus Europe initiative  Exercise as Medicine Campus recognition |
| 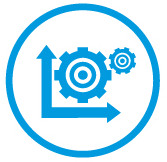 | **Adopt governance, management and budgetary structures to enable the vision** | Business plan founded on activity related income.  Governance structures developed to ensure quality and effectiveness in practice-based activity |

**Objectives of Physio Hub@UCD Sport**

- **Student Centred:** To support the student welfare mission of UCD Sport through the provision of health evaluations for the UCD Get in Gear Get Active student programme and musculoskeletal screening related to sports participation.
- **UCD Sports Teams and Clubs:** To support the musculoskeletal health, fitness and welfare of sports teams, sports club and sports scholars at UCD.
- **Educational Leader:** To facilitate UCD undergraduate and graduate student education in physiotherapy. This leverages existing clinical and academic staff resources from Physiotherapy.
- **Community Outreach:** To provide physiotherapy-led interventions for health, wellness and management of chronic disease to community dwelling adults, including but not limited to pathology specific group exercise programmes in association with expert practitioners.
- **Research Test-bed:** To embed the philosophy of research in practice through evaluation of the efficacy of Physio Hub activities and the investigation of related research questions.
